# The importance of using placebo controls in nonpharmacological randomised trials

**DOI:** 10.1097/j.pain.0000000000002839

**Published:** 2022-12-05

**Authors:** Karolina A. Wartolowska, David Hohenschurz-Schmidt, Lene Vase, Jeffrey K. Aronson

**Affiliations:** a Nuffield Department of Clinical Neurosciences, University of Oxford, John Radcliffe Hospital, Oxford, United Kingdom; b Pain Research, Department of Surgery and Cancer, Faculty of Medicine, Imperial College, Chelsea and Westminster Hospital Campus, London, United Kingdom; c Department of Psychology and Behavioural Sciences, School of Business and Social Sciences, Aarhus University, Aarhus, Denmark; d Centre for Evidence Based Medicine, Nuffield Department of Primary Care Health Sciences, Radcliffe Observatory Quarter, Oxford, United Kingdom

## 1. Introduction

Novel pharmacological compounds must undergo a series of highly regulated steps and have their efficacy demonstrated under strict conditions of placebo-controlled trials before being approved for clinical use.^17^ This is often not the case for treatments that do not involve a pharmacological element, such as surgery, physiotherapy, or psychological therapy. Despite the recognised need for evaluation,^31^ there are currently no formal requirements to test the efficacy of nonpharmacological medical procedures.

Failing to recognise that even large positive or negative effects may be caused by biases, rather than the medical properties of a treatment, may have serious consequences, as ineffective interventions may continue to be used, eg, spinal fusion for nonspecific back pain.^47^ Alternatively, effective treatments may be abandoned because negative effects are misattributed to the treatment.^26^

While some nonpharmacological treatments (Table 1), such as physiotherapy, are generally safe, and if they are not effective, the only harm may be a delay in providing effective therapy, others, such as surgery, are inherently associated with risks. If ineffective surgery continues to be used, not only does it waste time and resources, depriving patients of better treatment, but it also exposes patients to the risks associated with the procedure itself or anaesthesia, without any clinical benefits to justify them.

**Table 1:** Glossary of terms and explanation of fundamental concepts.

Term	Definition
Bias	A systematic distortion, due to a design problem, an interfering factor, or a judgement, that can affect the conception, design, or conduct of a study, or the collection, analysis, interpretation, presentation, or discussion of outcome data, causing erroneous overestimation or underestimation of the probable size of an effect or association
Allocation concealment	Allocation to the treatment or control groups done in a way that prevents preferential allocation of patients to a particular group
Blinding of patients	Concealment from patients of the treatment (active or control) to which they have been allocated. Blinding minimises many types of bias, including confounding, reporting bias, assessment bias, and bias caused by interactions with carers or clinical staff; it minimises dissatisfaction with the treatment allocation and the subsequent tendency to seek cotreatment; and it minimises drop-outs
Blinding of assessors	Concealment of the treatment allocation from those assessing and analysing the results. Assessor blinding may not be required if the outcomes are objective (eg, mortality, survival time, or laboratory tests) rather than subjective (eg, functional measures, quality-of-life scores, or other self-reported measures, including pain)
Blinding of providers	Concealment of the group allocation from those administering the treatment. In drug trials, this is typically done by overencapsulating or repackaging the medicines, but it is often challenging in nonpharmacological trials
Blinding—others	Blinding of other people involved in the trial. This prevents unintentional unblinding by revealing treatment allocation
Harm(s)	Adverse effects or adverse reactions, including nocebo effects; also delays to the introduction of effective treatment, propagation of harmful/unhelpful interventions/recommendations; generation of vested interests of providers into ineffective therapies; time and resources wasted on ineffective therapies
Nonpharmacological therapies	Nonpharmacological interventions are procedures that do not involve the use of a pharmacological agent; they include psychological and cognitive-behavioural approaches, exercise and rehabilitation, manual therapies, acupuncture, mind–body techniques such as yoga, devices such as ultrasound and light therapy, electrical therapies, education, and surgery; nutritional interventions are typically not included
Placebo control	A treatment or procedure ie, indistinguishable from the tested treatment, resembling it in all aspects but omitting the therapeutic components of the test treatment that are to be studied in the trial. In the case of surgery placebo control procedure may include anaesthesia/analgesia, standard care, and cointerventions
Randomisation	Random allocation to test and control group, which controls for small baseline differences between the groups and natural fluctuation of the studied condition
Test treatment	The intervention, or therapy the trial sets out to investigate
Treatment effect	The effect produced by the therapeutically active component of an intervention
Treatment provider	A person providing test and/or control treatment as part of a trial

In this topical review, we argue that not testing the efficacy of nonpharmacological procedures is problematic and should be addressed. We also outline possible steps to promote high-quality nonpharmacological efficacy trials.

## 2. Improvement and bias

Not all treatment effects are due to the clinical efficacy of the treatment; some arise from factors unrelated to the tested treatment.^21^ Some effects may be due to random error, ie, the play of chance, or to systematic error, also known as bias.

Bias refers to any systematic distortion causing erroneous overestimation or underestimation of the probable size of an effect or association^3^ (Tables 1 and 2). Biases in medicine are common, eg, those who receive a particular type of treatment may differ from those who receive another treatment or no treatment at all. The only way to minimise random error is to study many patients, ie, to have a sufficiently large sample size. However, strategies to minimise systematic errors depend on the type of bias, which is why different types of clinical trials are used.

**Table 2:** Sources of bias.

Bias may be caused by
Factors related to the investigated condition
Spontaneous symptomatic improvement or fluctuation
Factors related to the trial (including statistical phenomena)
Scaling bias caused by asymmetric rating scales
Poor definition of treatment efficacy
Irrelevant or surrogate outcome measures
Conditional switching of treatment
Ascertainment or selection bias
Regression to the mean
Confounding
Factors related to assessors
Observer bias
Training bias
Factors related to the patients themselves
Response bias
Acquiescence bias
Conditioning and expectancy effects
Interactions with doctors, including suggestions and information
The ritual of treatment
Misattribution
Apprehension bias or the Hawthorne effect

## 3. Elements of trial design

Well-designed clinical trials provide reliable and unbiased evidence of the efficacy of medical treatments. Studies aiming to demonstrate both the benefits and harms of treatments under highly controlled conditions are called efficacy trials.^38^ Their focus is on internal validity, achieved through minimising bias and standardising procedures to ensure that the treatment is implemented as intended.^25^ It is important to note that although well-designed trials^34^ can demonstrate both benefits and harms,^40^ trials are usually not powered to test harms.

### 3.1. Randomisation

In randomised controlled trials, patients are randomised, ie, randomly allocated to the tested treatment or the comparator, to reduce selection bias and the potential effect of nonrandom differences between the groups. Including a control arm allows estimation of regression to the mean or natural fluctuations during the course of a disease, which may be interpreted as evidence of a large effect.^15^

### 3.2. Comparator

The choice of the comparator or a control group depends on the aim of the trial and the potential biases. If the aim is to test whether a treatment is better than doing nothing, a no-treatment group or a waiting list may be used as a comparator. If the aim is to test relative efficacy, a tested treatment may be compared with an old treatment or an accepted “gold standard” treatment. For conditions that may only deteriorate and for so-called hard outcomes, ie, independent of judgement or assessment and robust to detection bias,^2^ comparative effectiveness trials may provide reliable results.

### 3.3. Placebo

A placebo is a treatment that is indistinguishable from the tested treatment but does not involve the key element of treatment expected to be responsible for the therapeutic effect that is being tested. If the main measures rely on patient reports, the outcomes may be biased by greater “emotional investment” and higher expectations^32^ in patients who know that they have been allocated to the tested treatment group because patients often believe that invasive,^24^ new,^44^ and expensive^43^ procedures are more effective. Conversely, the outcomes may be negatively biased by patients' dissatisfaction with allocation to a nontreatment control group.^13^ Without a placebo arm, interpretation of results may not be possible.^42^

A placebo arm is also useful to demonstrate reasons for improvement, related not to the crucial therapeutic element but to the therapeutic context or patient–treatment provider interactions. Although these effects have identifiable psychobiological mechanisms, they do not justify performing costly and potentially risky interventions if the therapeutic benefit is derived only from such context-dependent effects.

## 4. Challenges

Trials increasingly favour outcomes that prioritise patients' preferences and are based on patients' subjective reports. These outcomes are particularly prone to bias from lack of blinding^28^ and may require a placebo control. Using a placebo as a comparator is generally accepted in testing pharmacological treatments, to such an extent that “placebo pills” have become synonymous with placebos in general. Placebo-controlled trials of nonpharmacological treatments are equally important but less common. This is because of fewer regulatory requirements, misconceptions about sham controls, ethical problems, differences in the roles of treatments, and challenges of trial design and feasibility.

### 4.1. Misconceptions

A placebo in a pharmacological context is easy to comprehend; it is just, for example, a “sugar pill” or a saline injection, which is not associated with any inherent efficacy.^20^ In nonpharmacological trials, a “dummy drug” does not exist. The placebo comparator is often complex, and it is less clear which element represents the sham treatment and its delivery.

Moreover, in nonpharmacological trials, a placebo may involve varying degrees of invasiveness, each associated with potential risks. Although a placebo comparator tends to be safer than an active treatment, and involves the same precautions and analgesics as the tested intervention,^46^ patients may have misconceptions, eg, that placebo surgery means open surgery without any anaesthesia (personal communications after an IASP workshop).

### 4.2. False assumptions of efficacy

The “physical nature” of some nonpharmacological treatments, such as anatomical changes during surgery, the sensations produced by nerve stimulation, and the palpable nature of physical therapies,^24^ may lead to an assumption that these therapies are always effective, that their mechanisms of action are known, and that they do not need to be tested in clinical trials. However, for many nonpharmacological treatments, the exact mechanisms of action have not been convincingly proven. For example, narrowing of the subacromial space provides a mechanistically plausible explanation for shoulder impingement pain,^33^ but it is probably not the main mechanism because surgical tissue resection results in a similar improvement as arthroscopy without tissue removal^5^ or physiotherapy.^7,9^

### 4.3. Ethics

Efficacy trials are important because performing ineffective procedures, in which the chances of benefits do not balance out the risks of harms, is not ethical.^12^ In addition, recruiting patients into trials that cannot yield reliable results is not ethical; therefore, the trial design needs to be appropriate for the research question and the potential biases.^42^ From that perspective, placebo-controlled trials, even of invasive procedures, are justified because they may prevent continued use of a procedure without clinical benefits, saving time and resources that would otherwise be spent performing an ineffective procedure.^41^

The use of nonpharmacological placebo controls is ethically challenging. To be indistinguishable from the active treatment, the placebo comparator must involve some of the elements of the tested procedure—some of them associated with physical interactions and some risks of harm. Medical procedures are nowadays relatively safe,^16^ and placebos tend to be safer than active procedures^46^; however, any potential risks in the placebo arm make it less acceptable to patients and doctors. Patients worry about the potential adverse effects of placebo interventions,^8^ although they are willing to undergo the active intervention, even if a placebo-controlled trial has shown that it was risky and ineffective.^18^ Doctors are apprehensive about performing placebo procedures associated with risks,^45^ and anaesthetists oppose anaesthesia without clinical need.^8^

### 4.4. Treatment providers

Related to both the ethical and practical challenges of placebo-controlled nonpharmacological trials is the role of a treatment provider. Unlike drug trials in which a doctor prescribes a product manufactured by a pharmaceutical company, the treatment provider in nonpharmacological trials is personally and directly involved in delivering both the treatment and the placebo control.

Ethically, this creates a problem with equipoise because doctors have to simultaneously support the intervention sufficiently to perform it on their patients and doubt it sufficiently to trial it,^32^ which affects the acceptance of nonpharmacological placebos.^45^

From a practical perspective, blinding of treatment providers may not be possible because they are actively involved in performing the treatment as they operate, implant, or manipulate. Moreover, their expectations and allegiances may introduce biases.^30^

### 4.5. Recruitment and informed consent

One of the most significant ethical and practical challenges in trials is informed consent. The already elaborate consent process is more difficult and time-consuming when it involves recruitment into trials.^41^ Informing patients creates expectations, potentially resulting in placebo or nocebo effects, but not explaining the purpose of randomisation, and placebo controls may hinder recruitment into a trial.

Misconceptions about perceived efficacy, misunderstanding regarding placebo controls, and the complexity of placebo controls make the recruitment and consenting processes particularly difficult in nonpharmacological trials.^41^

### 4.6. Feasibility

Efficacy trials are challenging because of requirements for standardisation and minimisation of bias.^11^ This is even more of a problem for nonpharmacological trials because the equivalent of a dummy pill does not exist, identifying the key therapeutic element may be difficult, and standardising the test and control procedures is often challenging.^22^ This is further complicated by the cointerventions, which have to be standardised and potentially matched between the arms.

Because of the nature of many nonpharmacological interventions and the need for additional treatments, some trials cannot be blinded. When it is impossible to make a placebo control indistinguishable from the active procedure, a placebo-controlled trial should not be performed.^19^

### 4.7. Blinding

Outcomes that involve judgement, such as self-reported outcomes, assessments of radiological imaging, or clinical examinations can be prone to reporting and measurement bias when those rating or assessing outcomes are not blinded. However, even objective outcomes that involve no judgement (eg, mortality) may be biased in an unblinded study, owing to deviations in intended interventions where participants, researchers, and clinicians behave differently because of awareness of treatment allocation. Thus, blinding of participants through the use of a placebo as a comparator is still an important way to reduce bias, even when hard outcomes are used.^16,36^

Blinding of patients is intended to make the treatment and control condition more comparable, by balancing expectations between the conditions and reducing reporting bias in self-reported outcomes. Blinding of trial staff is also important to reduce measurement bias in any assessed outcomes that involve some form of judgement. Without adequate blinding, studies with subjective outcomes show larger effect sizes.^35^ Blinding of patients and all those involved in the trial and patient care also helps ensure that there will be no deviations from the intended intervention, including patients seeking additional treatments outside the trial and clinicians providing nonprotocol interventions. Finally, blinding of patients also reduces the likelihood of differences in drop-out rates between trial arms.

Confirming the success of blinding at the end of the trial by asking patients to guess their allocations has been criticised as testing participants' “hunches” about efficacy rather than the success of blinding, and it has been asserted that uncertainty about allocation is sufficient.^37^ However, despite controversies,^1,10^ testing blinding may improve a trial's internal validity—a null result in an adequately powered trial may suggest that blinding was maintained.

## 5. Potential solutions

### 5.1. Education

There is a great need to educate healthcare providers, patients, and the lay public about randomised clinical trials and placebo-controlled trials.^14^ The roles of different types of trials and the consequences of different biases need to be explained to doctors and other treatment providers during their training. It is also important to communicate, explain, and educate while approaching potential trial participants.

Careful choice of words is important from the very start when discussing nonpharmacological controls with patients and staff who are going to be involved.^8^ Although convenient, words such as “placebo,” “sham,” and “nonpharmacological” create certain assumptions that may be difficult to challenge later. It may be useful to avoid these words at the early stages of discussion about nonpharmacological placebos. A change of terminology would be useful, albeit challenging, but efforts are being made to improve it.^6^

### 5.2. Guidelines

The lack of evidence-based best-practice standards for development, implementation, and reporting of placebo control interventions is a major shortcoming. There is a great need for better reporting of adverse effects in trial publications^29^ and especially in trials of interventional procedures.^39^ However, recent guidelines on performing^4^ and reporting^23^ placebo controls are a notable improvement. More guidance related to specific nonpharmacological interventions is needed.

### 5.3. Financial incentives

Because there is no regulatory requirement for efficacy testing, and because procedures cannot be patented, there is less commercial interest, and less funding, for nonpharmacological trials.^22^ There is a place for public funding to support these types of trials, including financial support for allied health professionals who are involved in providing many types of nonpharmacological treatments.

There is a need for more funding for efficacy trials of nonpharmacological procedures, which would help overcome many feasibility problems and reduce treatment costs in the long term. Models of nonpharmacological therapies often use low-quality evidence to justify reimbursement, especially if there is public demand for the intervention. Sometimes more invasive and costly surgeries are chosen over less expensive physical therapies, despite a lack of strong evidence for better efficacy and lack of cost-effectiveness.^27^

## 6. Conclusions

Nonpharmacological interventions require unbiased evidence of safety and efficacy in the same way as pharmacological treatments. Randomised controlled trials of nonpharmacological interventions are relatively uncommon. Placebo-controlled trials, used to minimise bias in subjective outcomes, are even rarer, despite the popularity of patient-reported outcomes. The lack of efficacy trials of nonpharmacological procedures can be addressed by educating doctors and lay people, improving methodological guidance, introducing targeted funding, and using outcomes that do not require a placebo control arm.

## Conflict of interest statement

J. K. Aronson has written papers and edited textbooks on adverse drug reactions and has acted as an expert witness in legal and coroners’ cases involving such reactions. L. Vase has received consulting fees from Lundbeck D. Hohenschurz-Schmidt has received consulting fees from Altern Health Ltd. K. A. Wartolowska declares no conflicts of interest. This research has received no specific funding.
